# Supplementary material for: Experimental and Theoretical Approaches of New Nematogenic Chair Architectures of Supramolecular H-Bonded Liquid Crystals
Source: Molecules. 2020 Jan 16;25(2):365. doi: 10.3390/molecules25020365 (PMC7024265; doi:10.3390/molecules25020365)
Supplement: Supplementary file 1 [file molecules-25-00365-s001.pdf]

Supplementary data

Experimental and theoretical approaches of new nematogenic chair architectures of supramolecular H-bonded liquid crystals

O.A. Alhaddad[1], **H.A. Ahmed** [2,3*], **M. Hagar** [2,4*]

1. College of Sciences, Chemistry Department, Madina Monawara, Taibah University, Saudi Arabia, (OH) alhaddad105@yahoo.com.
2. College of Sciences, Chemistry Department, Yanbu, Taibah University, Saudi Arabia., (HA) ahoda@sci.cu.edu.eg, (MH) mohamedhaggar@gmail.com
3. Faculty of Science, Department of Chemistry, Cairo University, Cairo, Egypt.
4. Faculty of Science, Chemistry Department, Alexandria University, Alexandria, Egypt

* Correspondence: H.A. Ahmed, ahoda@sci.cu.edu.eg; M. Hagar, mohamedhaggar@gmail.com.

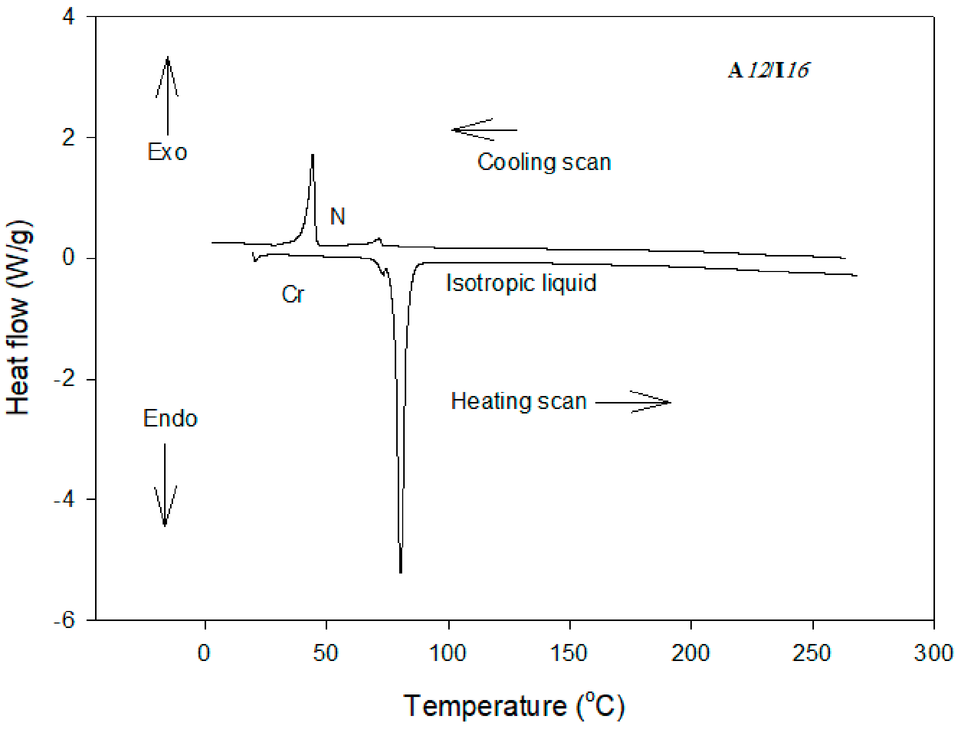

Figure S1. DSC thermograms of **A***12*/**I***16* supramolecular complex upon heating and cooling cycles with heating rate 10 oC/min.

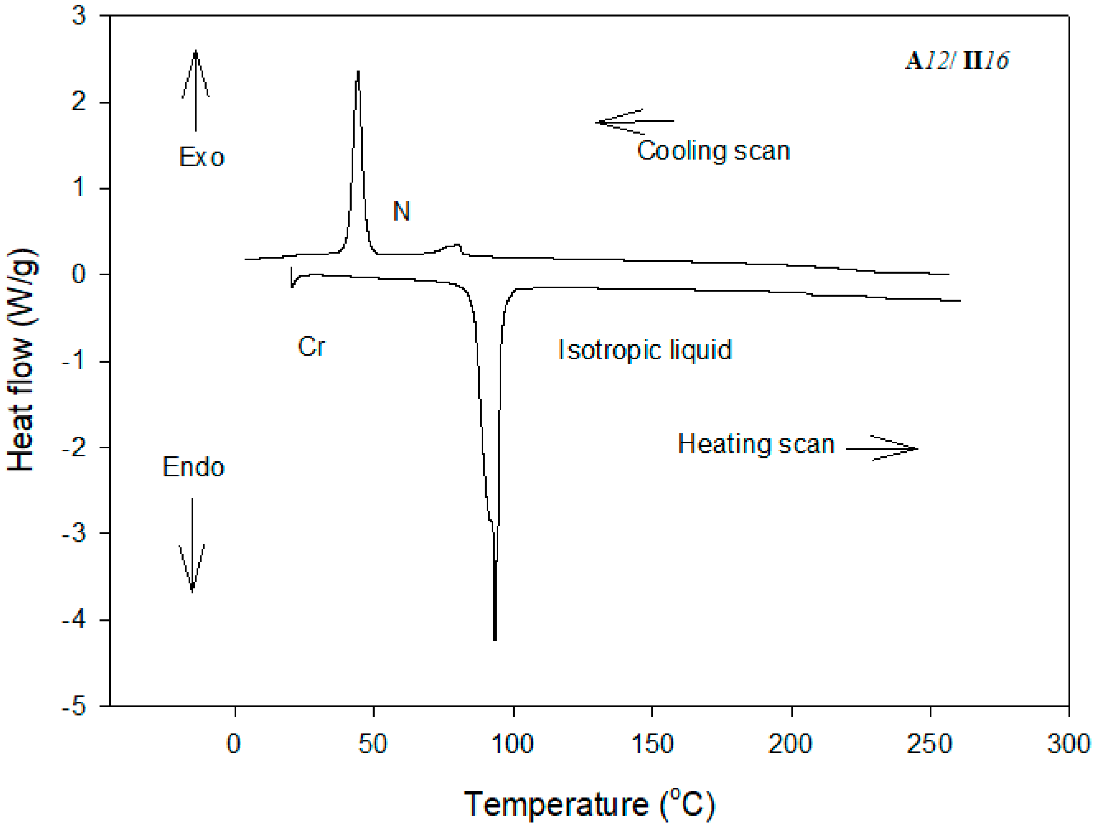

Figure S2. DSC thermograms of **A***12*/**II***16* supramolecular complex upon heating and cooling cycles with heating rate 10 oC/min.

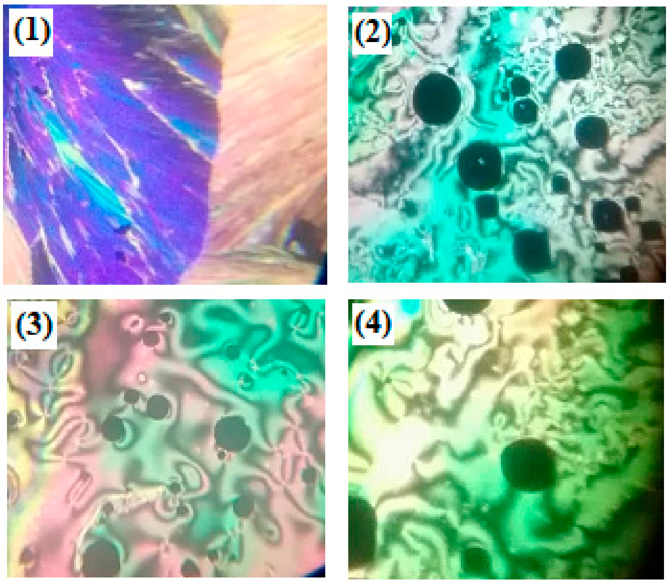

Figure S3: Some textures (Size: 41.8x 10^6 nm) under POM of the supramolecular complexes upon heating (1) solid phase of **A**10/**I**8 at 52.0 ºC; (2) nematic phase of **A**6/**I**8 at 75.0 ºC; (3) nematic phase of **A**6/**II**8 at 112.0 ºC; and (4) nematic phase of **A**8/**II**8 at 85.0 ºC.

Table S1: Normalized entropy change (Δ***S***/R) for the supramolecular complexes **A***n*/**III***16* and **A***n*/**IV***16*.

System	Δ***S****/R*
**A**6/**III***16*	1.18
**A**8/ **III***16*	1.42
**A***10*/ **III***16*	2.27
**A***12*/ **III***16*	2.40
**A**8/**IV***16*	0.50
**A***10*/ **IV***16*	0.41
**A***12*/ **IV***16*	2.22
**A***14*/ **IV***16*	2.19
